# Epigenetic Regulators as Therapeutic Targets in Pancreatic Ductal Adenocarcinoma

**DOI:** 10.3390/cancers18061001

**Published:** 2026-03-19

**Authors:** Klaudia Kubiak, Iwona Inkielewicz-Stępniak

**Affiliations:** Department of Pharmaceutical Pathophysiology, Medical University of Gdansk, 80-211 Gdansk, Poland; iwona.inkielewicz-stepniak@gumed.edu.pl

**Keywords:** pancreatic ductal adenocarcinoma (PDAC), epigenetics, DNA methylation, histone modification, chromatin remodeling, non-coding RNA, epigenetic therapy

## Abstract

Pancreatic ductal adenocarcinoma (PDAC) remains one of the most lethal malignancies, driven by aggressive tumor biology, extensive intratumoral heterogeneity, and profound resistance to standard therapies. While recurrent genetic alterations such as KRAS mutations are central to PDAC initiation, growing evidence demonstrates that epigenetic dysregulation is a critical determinant of disease progression, cellular plasticity, immune evasion, and therapeutic failure. Epigenetic mechanisms, including DNA methylation, histone modifications, chromatin remodeling, and non-coding RNA regulation, shape transcriptional programs without altering the underlying DNA sequence, rendering them dynamic and potentially reversible therapeutic targets. This review provides a comprehensive overview of key epigenetic proteins implicated in PDAC, encompassing writers, readers, and erasers of chromatin marks. Aberrant activity of histone methyltransferases and acetyltransferases, bromodomain-containing proteins, histone deacetylases, and demethylases orchestrates transcriptional reprogramming that promotes epithelial–mesenchymal transition, stem-like phenotypes, metabolic adaptation, and resistance to chemotherapy and radiotherapy. In parallel, epigenetic alterations within the tumor microenvironment contribute to stromal activation and immune suppression, further limiting therapeutic efficacy. We summarize recent advances in pharmacological targeting of epigenetic regulators and discuss the rationale for combination strategies integrating epigenetic inhibitors with cytotoxic agents, targeted therapies, and immunotherapies. Emphasis is placed on emerging experimental platforms—including patient-derived organoids, co-culture systems, and in vivo models—combined with multi-omic profiling and computational approaches to identify biomarkers of response and optimize therapeutic design. Collectively, this review highlights epigenetic regulation as a central and actionable vulnerability in PDAC and outlines future directions toward biomarker-guided, personalized epigenetic therapies aimed at overcoming resistance and improving clinical outcomes.

## Simple Summary

Pancreatic ductal adenocarcinoma is one of the deadliest cancers, largely due to late diagnosis and resistance to available treatments. Beyond genetic mutations, cancer cells are strongly influenced by epigenetic mechanisms—reversible chemical modifications that control gene activity without changing the DNA sequence. In pancreatic cancer, these epigenetic alterations reshape tumor behavior, promote spread, and enable resistance to therapy. This review summarizes current knowledge on key epigenetic proteins that regulate chromatin structure and gene expression in pancreatic cancer, including enzymes that write, read, or erase epigenetic marks. We also discuss emerging drugs targeting these regulators and highlight how combining epigenetic therapies with existing treatments may improve therapeutic outcomes. By integrating molecular biology, computational approaches, and preclinical models, this work aims to provide a framework for translating epigenetic discoveries into more effective diagnostic and therapeutic strategies for pancreatic cancer.

## 1. Introduction

Pancreatic cancer, predominantly pancreatic ductal adenocarcinoma (PDAC), is among the most aggressive solid malignancies and is projected to become the second leading cause of cancer-related mortality by 2040 [1,2]. Surgical resection remains the only potentially curative treatment; however, only 10–15% of patients present with resectable disease at diagnosis [3]. Approximately 30–35% of patients are diagnosed with locally advanced unresectable disease and nearly 50% with metastatic disease, both associated with a dismal prognosis [3]. Despite advances in systemic therapy, including combination chemotherapy regimens such as FOLFIRINOX and gemcitabine plus nab-paclitaxel, median overall survival for advanced PDAC remains approximately 8–12 months [4,5,6,7].

In recent years, emerging therapeutic strategies, including molecularly targeted agents, immunotherapy, and rational combination approaches, have demonstrated modest yet clinically meaningful activity in molecularly defined patient subsets. Reflecting this progress, updated American Society of Clinical Oncology (ASCO) guidelines now incorporate molecularly guided treatment options for selected PDAC subgroups [6]. Nevertheless, the overall clinical impact of precision oncology in PDAC remains constrained by profound biological complexity, extensive intratumoral heterogeneity, and the limited prevalence of actionable alterations.

A well-established example of precision therapy in PDAC is the use of platinum-based chemotherapy in patients harboring germline mutations in homologous recombination repair genes such as *BRCA1*, *BRCA2*, or *PALB2*. Exploiting this vulnerability, maintenance therapy with the poly(ADP-ribose) polymerase (PARP) inhibitor olaparib induces synthetic lethality in BRCA-deficient tumors [8] and has shown clinical benefit in the phase III POLO trial [9]. Accordingly, PARP inhibition is now considered a clinically relevant maintenance strategy following platinum-based chemotherapy in eligible patients.

Another targeted approach entering clinical practice involves immune checkpoint inhibition in tumors with DNA mismatch repair deficiency (dMMR) or high microsatellite instability (MSI-high). Pembrolizumab, an anti–PD-1 antibody, has received tissue-agnostic FDA approval for dMMR/MSI-high malignancies [10]. Although this approval includes PDAC, the prevalence of dMMR/MSI-high tumors in this disease is low (approximately 1–2%), and evidence supporting robust and durable clinical responses remains limited [11,12].

A major recent breakthrough in PDAC therapy has been the successful pharmacological targeting of mutant KRAS. The KRAS^G12C inhibitor sotorasib demonstrated antitumor activity in PDAC, providing proof-of-concept that direct KRAS inhibition is feasible despite decades of perceived “undruggability” [13,14,15]. However, KRAS^G12C mutations occur in only 1–2% of PDAC cases. More recently, the KRAS^G12D-selective inhibitor MRTX1133 has shown pronounced antitumor efficacy in preclinical PDAC models [16,17], particularly relevant given that KRAS^G12D represents the most prevalent KRAS mutation, occurring in approximately 40% of patients [18]. Despite these advances, substantial clinical validation is still required before KRAS-directed therapies can be broadly implemented in routine practice.

Collectively, these examples underscore that precision oncology remains the exception rather than the rule in PDAC. Comprehensive molecular profiling is not yet universally implemented in clinical practice, and currently approved targeted therapies benefit only small, molecularly defined subsets of patients. This limited therapeutic landscape highlights the urgent need to expand the repertoire of actionable vulnerabilities and to better understand the regulatory mechanisms that drive PDAC progression, phenotypic heterogeneity, and therapy resistance.

Genomic studies have identified four core driver genes, *KRAS*, *TP53*, *CDKN2A*, and *SMAD4*, that are mutated in the majority of PDAC cases. Beyond these recurrent alterations, however, few additional mutations occur at frequencies exceeding 5% [19]. Integrative genomic analyses have instead revealed recurrent pathway-level perturbations, including alterations in histone-modifying enzymes (e.g., *KDM6A*), SWI/SNF chromatin remodeling complexes (e.g., *ARID1A*), and DNA damage repair pathways (e.g., *BRCA1/2*) [20]. Apart from homologous recombination–deficient tumors, most of these molecular alterations have not yet been successfully translated into effective therapeutic strategies. Importantly, the mutational landscape alone fails to fully explain the extensive interpatient and intratumoral heterogeneity characteristic of PDAC.

Indeed, PDAC phenotypic diversity is more accurately reflected at the transcriptomic level than by genomic alterations alone. Transcriptomic profiling has consistently identified two major prognostically relevant subtypes: the well-differentiated “classical” subtype, characterized by epithelial gene expression programs and comparatively improved survival, and the poorly differentiated, invasive, and chemoresistant “basal-like” subtype, associated with adverse clinical outcomes [21,22,23,24,25]. While specific genetic features, such as increased KRAS dosage, have been linked to subtype identity [21], most tumors harbor an overlapping spectrum of core driver mutations. These observations indicate that non-genetic mechanisms play a central role in establishing and maintaining distinct transcriptional states.

**Figure 1:**
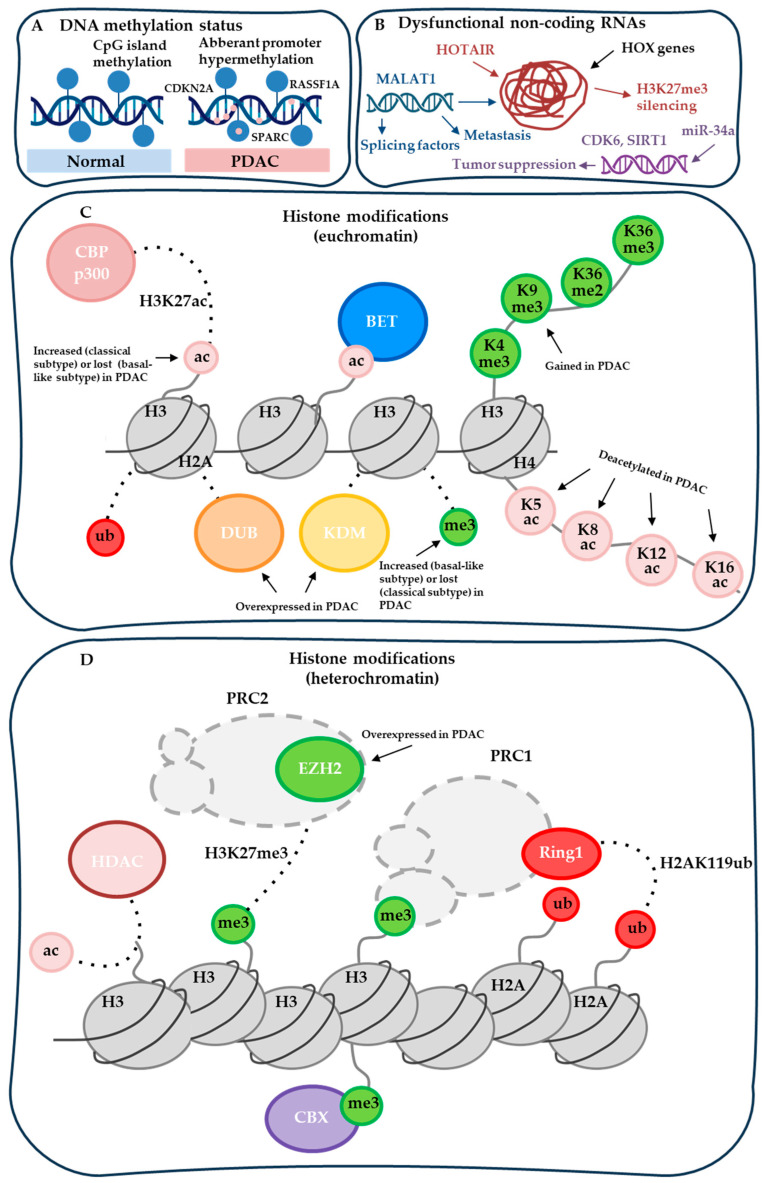
Schematic overview of epigenetic dysregulation in PDAC. The figure illustrates major epigenetic mechanisms contributing to transcriptional dysregulation and disease progression in PDAC. (**A**) DNA methylation dysregulation: In normal cells, CpG islands at promoters are unmethylated, allowing active transcription of tumor-suppressor genes. In PDAC, aberrant promoter hypermethylation silences key tumor suppressors (e.g., CDKN2A/p16, SPARC, RASSF1A), promoting proliferation and invasion. Concurrent global hypomethylation activates repetitive elements, drives genomic instability, and contributes to oncogenic transcription. These changes reinforce basal-like/squamous subtype identity and chemoresistance. (**B**) Non-coding RNA involvement: Oncogenic lncRNAs (HOTAIR recruits PRC2 for H3K27me3 deposition and gene silencing; MALAT1 modulates splicing and chromatin looping) promote EMT, metastasis, and stemness. Tumor-suppressive miRNAs (miR-34a) are frequently silenced by promoter methylation, leading to unchecked Notch/Snail signaling and progression. (**C**) Transcriptionally active (euchromatic) states: Histone acetyltransferases (HATs) (e.g., CBP/p300) add acetyl groups (H3K27ac, H4Kac) at enhancers/promoters, recognized by BET proteins to recruit transcriptional machinery. Activating methylation marks (H3K4me3, H3K36me3) are maintained by writers; lysine demethylases (KDMs) (KDM6A/UTX removes repressive H3K27me3 at enhancers; KDM1A/LSD1 demethylates H3K4me at poised states) and deubiquitinases (DUBs) (USP22 removes H2Bub1 to facilitate elongation) actively sustain open chromatin for epithelial gene programs in classical PDAC subtypes. (**D**) Transcriptionally repressed (heterochromatic) states: Polycomb repressive complexes (PRC2 via EZH2 deposits H3K27me3; PRC1 adds H2AK119ub) compact chromatin and silence differentiation genes (CDH1/E-cadherin, CDKN1A/p21), driving dedifferentiation, EMT, and basal-like phenotypes in PDAC. Histone deacetylases (HDACs) remove acetyl groups, reinforcing repression. These alterations enable rapid adaptation to therapeutic stress, subtype plasticity, immune evasion, and stromal remodeling in PDAC. Figure concept courtesy of Maria Wiese, Department of Pediatrics and Adolescent Medicine, Medical University of Göttingen, Germany. This figure was created with BioRender.com.

Epigenetic regulation has emerged as a key mechanism underlying PDAC plasticity, subtype specification, heterogeneity, and adaptive resistance. Epigenetic alterations are defined as heritable yet potentially reversible changes in gene expression that occur without modifications of the underlying DNA sequence. These include DNA methylation, post-translational histone modifications, chromatin remodeling, and regulation by non-coding RNAs (Figure 1). Through dynamic modulation of chromatin architecture, epigenetic mechanisms govern transcriptional programs controlling cellular differentiation, stemness, metastatic potential, and therapeutic response. Unlike fixed genetic mutations, epigenetic alterations are potentially reversible, positioning epigenetic regulators as attractive therapeutic targets.

Mounting evidence indicates that dysregulation of epigenetic proteins, commonly categorized as “writers,” “erasers,” “readers,” and chromatin remodelers, plays a critical role in PDAC initiation, progression, subtype determination, and treatment resistance. By regulating transcriptional identity independently of DNA sequence alterations, epigenetic mechanisms enable tumor cells to rapidly adapt to environmental and therapeutic pressures, thereby contributing to disease aggressiveness and poor clinical outcomes. Consequently, targeting epigenetic regulators represents a promising strategy to overcome tumor plasticity, enhance therapeutic sensitivity, and expand precision medicine approaches in PDAC. Importantly, these transcriptional subtypes are not merely descriptive classifications but reflect distinct epigenetic dependencies. Basal-like tumors exhibit enhanced Polycomb Repressive Complex 2 (PRC2) activity, super-enhancer remodeling, and increased chromatin plasticity, rendering them potentially more sensitive to Enhancer of zeste homolog 2 (EZH2) or Bromodomain and extraterminal domain (BET) inhibition. In contrast, classical tumors maintain enhancer-driven epithelial programs that may depend on CBP/p300 (CREB-binding protein/E1A binding protein p300)-mediated acetylation. This subtype-specific epigenetic wiring suggests that therapeutic targeting should be stratified accordingly.

While epigenetic dysregulation has long been recognized as a key driver of PDAC biology beyond genetic mutations, recent reviews have advanced our understanding of specific facets. For example, Pandey et al. (2023) provided a broad overview of epigenetic machinery components in PDAC initiation and development, while Elrakaybi et al. (2022) and Sultana et al. (2025) highlighted epigenetic impacts on biology, diagnostics (methylation-based biomarkers), and emerging clinical applications [26,27,28]. More focused works include those by Tost et al. (2025), on leveraging epigenetic alterations for prognostic and therapeutic insights, Maietta et al. (2025), on harnessing epigenetic inhibitors to overcome PDAC treatment challenges, and Drndakova et al. (2026), on epigenetic modulation to reverse immune suppression in the tumor microenvironment [29,30,31]. Earlier contributions, such as methylation-centric analyses [32,33] and histone-focused or therapeutic overviews [34], laid the foundational knowledge.

Despite these valuable syntheses, the present review offers distinct advantages: (i) a comprehensive, integrated framework encompassing all major epigenetic regulator classes (writers, readers, and erasers of DNA methylation and histone modifications) in a unified manner; (ii) dedicated emphasis on the tumor microenvironment as an epigenetically regulated compartment, including stromal reprogramming and immune evasion; (iii) synthesis of cutting-edge experimental platforms (patient-derived organoids, co-culture systems) combined with multi-omic profiling and computational approaches for mechanistic and translational insights; and (iv) a forward-looking precision medicine perspective prioritizing biomarker-guided, subtype-specific (classical vs. basal-like) epigenetic therapeutic stratification to overcome resistance and improve outcomes. By bridging these elements, this work aims to guide future biomarker-driven and combination epigenetic strategies in PDAC.

## 2. DNA Methylation

DNA methylation, most commonly the covalent addition of a methyl group to cytosine within CpG dinucleotides, is a fundamental epigenetic mechanism regulating gene expression through modulation of chromatin accessibility and transcription factor binding at promoters and enhancers. Promoter CpG island hypermethylation represents a canonical mechanism of stable gene silencing, including inactivation of tumor suppressor genes, whereas focal and global hypomethylation contributes to transcriptional dysregulation, chromosomal instability, and activation of repetitive elements in cancer [35].

Recent integrative analyses combining whole-genome bisulfite sequencing (WGBS) with transcriptomic profiling have demonstrated that DNA methylation landscapes closely parallel PDAC transcriptional subtypes. Classical tumors generally retain epithelial lineage programs and exhibit relatively preserved promoter methylation at lineage-defining transcription factors such as *GATA6*, whereas basal-like tumors display extensive promoter and enhancer hypermethylation at epithelial regulators together with hypomethylation at loci associated with inflammatory and mesenchymal programs [21,22,23,24,25,36]. Importantly, these subtype-specific methylation patterns not only reflect underlying transcriptional states but also contribute to their stabilization, suggesting that targeted remodeling of the methylome could potentially reprogram aggressive PDAC phenotypes.

### 2.1. Aberrant Methylation in PDAC

Aberrant DNA methylation is an early and pervasive event in PDAC tumorigenesis. Genome-wide methylome analyses, including WGBS of primary tumors and matched organoid models, reveal stage- and subtype-specific reprogramming during disease progression. Advanced tumors exhibit large-scale methylome alterations, and squamous/basal-like transcriptional programs are frequently associated with distinct promoter and enhancer hypermethylation patterns affecting epithelial regulatory networks, including *GATA6* [36]. These findings support a model in which DNA methylation both tracks and reinforces transcriptional subtype identity.

Writers such as DNMT1 (overexpressed in ~80% of cases) maintain aberrant hypermethylation at promoters of tumor suppressors (CDKN2A, SPARC, RASSF1A), driving silencing and aggressive phenotypes including chemoresistance [35]. De novo writers DNMT3A/B further establish oncogenic methylation patterns [37]. Erasers like TET1 are frequently downregulated, reducing 5hmC levels and failing to activate Wnt/β-catenin or Hedgehog inhibitors, thereby promoting EMT and metastasis [38]. Readers including MBD proteins (MBD1 repressing KEAP1 to activate NRF2 survival signaling) [39], MeCP2 (mediating repressive chromatin compaction) [40], UHRF1 (recruiting DNMT1 and modulating metabolism via SIRT4 repression), and Kaiso (context-dependent methylation reader) [41] translate these marks into transcriptional repression or activation, contributing to immune evasion, metabolic adaptation, and stromal interactions. Therapeutic targeting focuses on DNMT inhibitors to reverse hypermethylation, with emerging strategies to restore TET activity or disrupt reader complexes, often in combinations to enhance efficacy and overcome resistance [42].

### 2.2. Diagnostic and Circulating Biomarkers

Aberrant DNA methylation represents one of the most promising classes of epigenetic biomarkers for PDAC, detectable non-invasively in circulating cell-free DNA (cfDNA) from blood (liquid biopsy) or pancreatic juice. Genome-wide and targeted methylation profiling has identified tumor-specific hypermethylation at promoters of tumor suppressor genes (CDKN2A, SPARC, RASSF1A, APC, BNCI) and global hypomethylation contributing to genomic instability, patterns that distinguish PDAC from normal pancreas, benign lesions, or chronic pancreatitis [43,44,45,46].

cfDNA-based methylation assays offer several key advantages over traditional diagnostics. Their non-invasive nature enables serial sampling for longitudinal monitoring of disease progression, treatment response, or recurrence without repeated invasive procedures [47,48]. Methylation signatures can detect aberrant epigenetic changes earlier than imaging or the serum marker CA19-9 alone, as hypermethylation often precedes gross tumor formation and detectable protein elevations [49,50]. Liquid biopsy captures tumor heterogeneity by sampling shed DNA from multiple subclones, potentially outperforming single-site tissue biopsies in heterogeneous PDAC [26] [Sultana et al., 2025]. Multi-marker panels (combining 3–13 methylated loci like FER1L4, ADAMTS1, BNC1 with CA19-9) achieve high specificity (88–97%) and improved sensitivity (83–89% for all stages; 83% for stage I/II in some cohorts), providing complementarity to protein markers (CA19-9) and ctDNA mutation profiling (KRAS) [48,49,50]. Emerging 5-hydroxymethylcytosine (5hmC) profiles in cfDNA further enhance early-stage detection by reflecting active demethylation loss, with AUCs of 0.92–0.94 in validation sets [48]. These features position methylation-based liquid biopsy as a tool for high-risk screening (familial PDAC, new-onset diabetes) and minimal residual disease monitoring.

Despite the promise, significant hurdles limit clinical translation. Sensitivity remains relatively low for early-stage (I/II) disease, where circulating tumor-derived DNA is sparse (<1–10 copies/mL blood), yielding detection rates of 60–70% even in optimized panels (61.9–68.3% for stage I/II in multi-cancer tests) [48,50]. This contrasts with higher sensitivity in advanced stages (85–95%) but underscores challenges in asymptomatic or pre-invasive detection. Inter-laboratory variability arises from differences in bisulfite conversion efficiency, sequencing depth, bioinformatic pipelines (DMR calling), and normalization methods, hindering reproducibility [45,47]. Large prospective validation cohorts in screening-relevant populations (high-risk asymptomatic individuals) are scarce, with most data from case–control studies prone to bias [43]. False positives can occur from age-related, inflammatory (pancreatitis), or smoking-induced methylation changes unrelated to malignancy [44]. Finally, standardized clinical-grade platforms are lacking—no FDA-approved PDAC-specific methylation assay exists, and integration into routine practice requires harmonized protocols, cost-effectiveness data, and regulatory validation [30].

To maximize potential, methylation signatures should be integrated with orthogonal modalities (multi-omic panels combining methylation, fragmentomics, 5hmC, ctDNA mutations, and proteins like TIMP1/LRG1) for synergistic performance [50]. Machine learning-optimized algorithms and prospective trials in high-risk cohorts will be essential to refine sensitivity/specificity and establish clinical utility. Ultimately, cfDNA methylation holds transformative promise for shifting PDAC diagnosis toward earlier, curable stages, but requires rigorous validation to overcome current barriers.

### 2.3. Therapeutic Targeting of DNA Methylation

**Figure 2:**
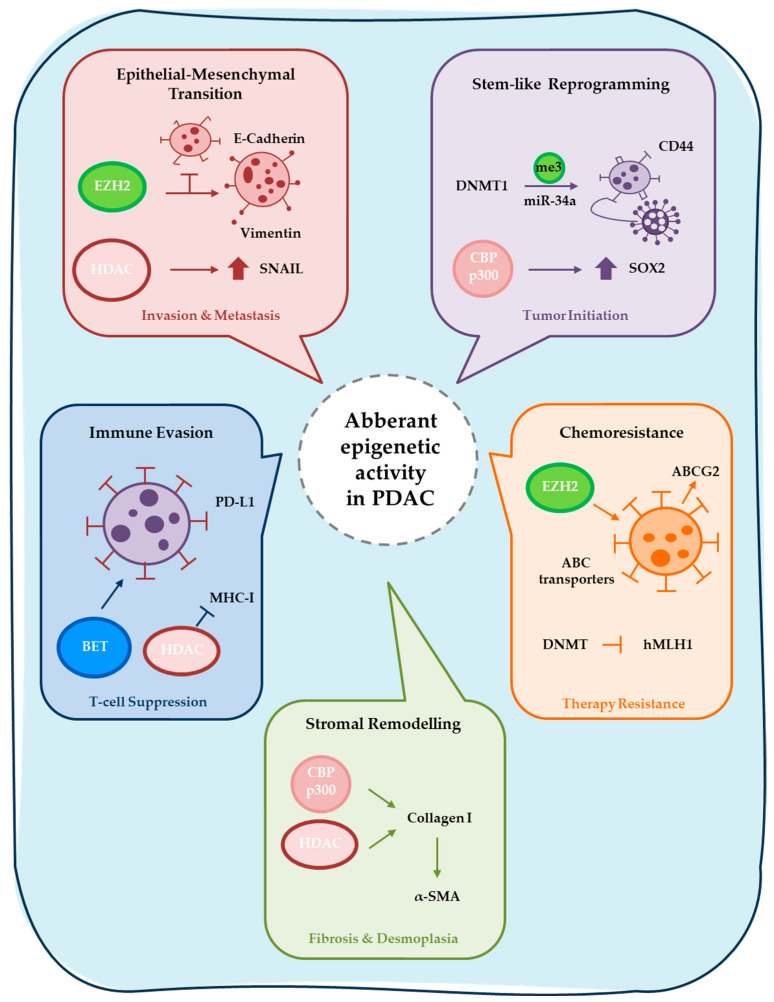
Epigenetic dysregulation drives key oncogenic hallmarks in pancreatic ductal adenocarcinoma (PDAC). Schematic overview depicting how aberrant activity of major epigenetic regulators contributes to PDAC progression and therapy resistance. Core oncogenic hallmarks in PDAC (epithelial–mesenchymal transition [EMT], stem-like reprogramming, immune evasion, chemoresistance, and stromal remodeling in the tumor microenvironment [TME]) are interconnected and promoted by dysregulated epigenetic machinery. EZH2/PRC2: Overexpression leads to H3K27me3 deposition, silencing epithelial genes (CDH1/E-cadherin, miR-200 family), thereby promoting EMT and invasion/metastasis. EZH2 also contributes to stemness and chemoresistance. HDACs: Deacetylate histones (loss of H3/H4 acetylation), repress tumor suppressor promoters (CDKN2A, SPARC), activate EMT transcription factors (SNAIL), suppress MHC-I expression (immune evasion), and enhance stromal activation via CAF remodeling. DNMTs: Promoter hypermethylation silences tumor suppressors and DNA repair genes (hMLH1), while also methylating miR-34a, promoting stem-like properties (SOX2, NANOG activation) and chemoresistance via ABC transporters. BET proteins: Read acetylated histones, drive super-enhancer-mediated transcription of oncogenes (c-MYC, BCL2), upregulate PD-L1 expression (immune evasion), and support survival/anti-apoptotic programs. CBP/p300: Acetylate H3K27, activates stemness genes (SOX9) and stromal collagen production, contributing to desmoplasia and TME remodeling; also involved in repair gene activation in certain contexts. Arrows indicate activating (→) or repressive (⊣) influences. This figure was created with BioRender.com.

The nucleoside analog DNA methyltransferase inhibitors (DNMTis), azacitidine and decitabine, are clinically approved hypomethylating agents that incorporate into DNA and inhibit DNMT activity, leading to passive demethylation and re-expression of silenced genes (Figure 2). In PDAC, early-phase clinical trials evaluating DNMTi as monotherapy or in combination with chemotherapy or immune checkpoint inhibitors have demonstrated pharmacodynamic activity, including partial demethylation and induction of immune-related genes in subsets of patients. However, durable clinical responses in unselected PDAC populations have been limited [51,52,53,54,55]. Several biological and pharmacological factors contribute to these modest outcomes. The dense desmoplastic stroma characteristic of PDAC impairs intratumoral drug penetration, particularly for short half-life nucleoside analogs. High expression of cytidine deaminase in pancreatic tissue accelerates metabolic inactivation of DNMTi, reducing effective drug exposure and necessitating higher doses that may increase systemic toxicity. Furthermore, remethylation following treatment discontinuation can rapidly attenuate pharmacodynamic effects. Finally, the absence of validated methylation-based predictive biomarkers has resulted in the treatment of molecularly unselected patient populations, likely diluting therapeutic benefit.

Combination strategies have therefore been explored to enhance efficacy. Clinical trials have evaluated azacitidine in combination with pembrolizumab and decitabine with gemcitabine, demonstrating acceptable tolerability but limited efficacy signals in unselected cohorts [56,57]. Ongoing translational efforts focus on defining pharmacodynamic endpoints, such as depth and durability of demethylation and induction of immune-related gene signatures, to refine dosing schedules and improve patient selection.

To overcome limitations of first-generation DNMTi in PDAC, several strategies are under investigation: (i) development of novel DNMTi chemotypes and oral formulations with improved metabolic stability; (ii) co-administration of cytidine deaminase inhibitors or use of CDA-resistant prodrugs to enhance systemic exposure; (iii) nanoparticle-based or stroma-penetrating delivery systems to improve tumor uptake; and (iv) rational biomarker-guided combinations exploiting DNMTi-induced immune priming (e.g., sequential DNMTi followed by anti–PD-1/PD-L1 therapy) or synergy with PARP inhibitors and chemotherapy in defined molecular contexts. DNMTi-mediated demethylation can reactivate silenced genes involved in homologous recombination (HR) repair and promote replication stress through the induction of endogenous retroelement expression and dsRNA sensing. Simultaneously, PARP inhibitors exploit HR deficiency to trap PARP1/2 at single-strand breaks, inducing cytotoxic double-strand breaks. The combination is therefore mechanistically synergistic in HR-deficient contexts: DNMTi may sensitize tumors by further impairing HR capacity through epigenetic reprogramming, while also inducing immune priming via viral mimicry. In PDAC, this combination is particularly relevant in BRCA1/2- or PALB2-mutant tumors, and in tumors with epigenetically silenced HR genes, where DNMTi-induced re-expression of HR factors may paradoxically need to be avoided to preserve PARP inhibitor efficacy.

Collectively, preclinical and early clinical data support continued development of methylation-targeted strategies in PDAC. However, clinical success will likely depend on improved pharmacological properties, stromal delivery optimization, and robust biomarker-driven patient stratification.

## 3. Histone Modifications

**Figure 3:**
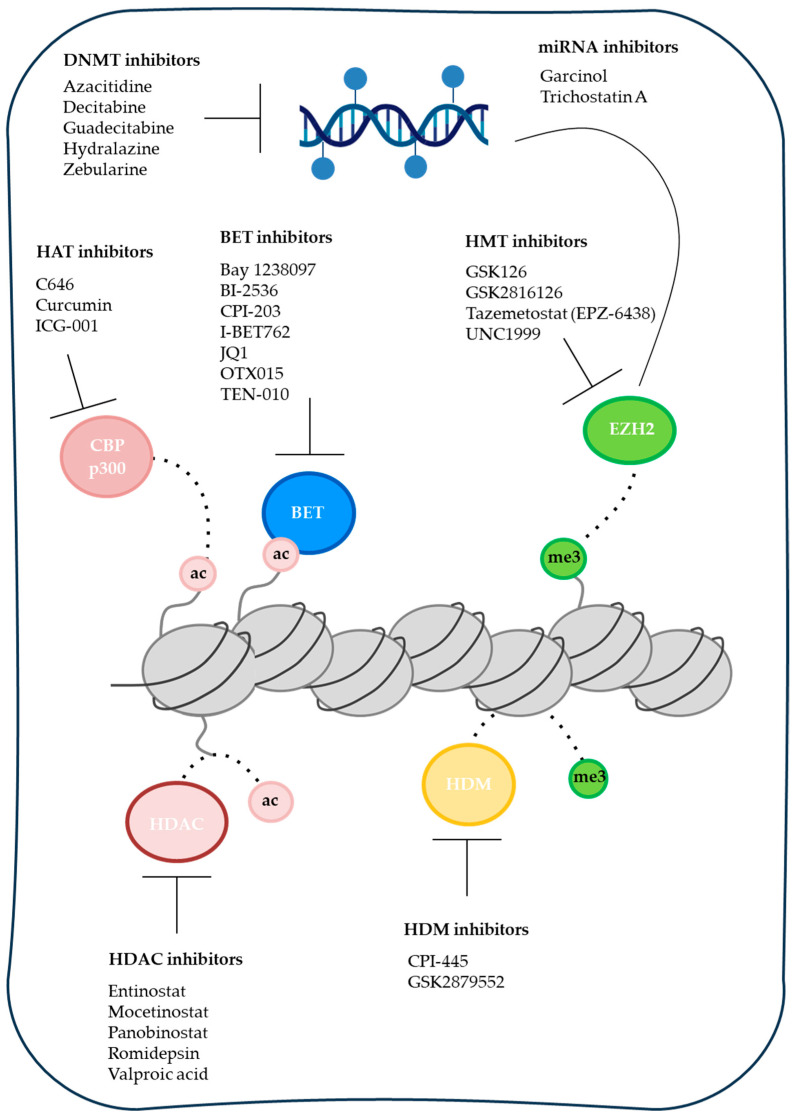
Overview of epigenetic drugs investigated in preclinical studies and clinical trials for PDAC treatment. The figure summarizes first-, second-, and third-generation epigenetic inhibitors targeting diverse chromatin regulators that have been investigated in preclinical models and clinical trials of PDAC. While epigenetic drug monotherapies have shown limited efficacy, combination strategies targeting multiple epigenetic mechanisms or biomarker-driven approaches integrating next-generation epigenetic agents with conventional therapies currently represent the most promising therapeutic concepts for PDAC. The figure was created using BioRender.com.

Chromatin regulation by histone post-translational modifications (PTMs) constitutes a fundamental epigenetic mechanism controlling gene expression programs in development and cancer. Histone PTMs are dynamically installed, recognized, and removed by epigenetic writers, readers, and erasers, which collectively regulate chromatin accessibility and transcription factor recruitment (Figure 3) [58,59,60]. These enzymes catalyze or interpret covalent modifications on histone tails, including methylation, acetylation, ubiquitination, and phosphorylation, thereby enabling context-dependent transcriptional regulation [61,62].

The functional consequences of histone PTMs depend on genomic context, modification state (mono-, di-, or trimethylation), and combinatorial interactions with DNA methylation and chromatin remodeling complexes [63]. Disruption of this tightly regulated network is a hallmark of malignant transformation and contributes to tumor initiation, lineage plasticity, metastasis, and therapy resistance. In PDAC, oncogenic KRAS signaling drives extensive epigenomic reprogramming, reshaping enhancer landscapes and histone modification patterns to sustain proliferative, metabolic, and stress-adaptive transcriptional states [64,65,66]. These findings position histone-modifying enzymes as key mediators of PDAC transcriptional identity and therapeutic vulnerability.

### 3.1. Epigenetic Writers

#### 3.1.1. EZH2 and the Polycomb Repressive Complex 2 (PRC2)

EZH2 is the catalytic subunit of PRC2 and mediates trimethylation of histone H3 at lysine 27 (H3K27me3), a repressive chromatin mark associated with stable gene silencing [67]. In PDAC, EZH2 is frequently overexpressed and correlates with high tumor grade, epithelial–mesenchymal transition (EMT), metastatic dissemination, and reduced survival [68,69,70].

Mechanistically, EZH2 represses differentiation- and cell cycle–regulatory genes, including *CDKN1A (p21)* and *CDH1 (E-cadherin)*, thereby promoting dedifferentiation, proliferation, and invasive behavior [71,72]. H3K27me3 deposition facilitates recruitment of PRC1 complexes, whose RING1A/B subunits catalyze H2AK119 monoubiquitination, reinforcing chromatin compaction and transcriptional repression [73].

Selective EZH2 inhibitors such as tazemetostat (EPZ-6438) and GSK126 reactivate silenced tumor suppressor genes, suppress proliferation, and reduce metastatic capacity in PDAC cell lines, organoids, and xenograft models [74,75,76]. Importantly, EZH2 inhibition enhances sensitivity to gemcitabine and synergizes with HDAC or BET inhibition, highlighting functional cooperation between epigenetic pathways. However, PRC2 function in PDAC appears context-dependent. In genetically defined settings, such as *KDM6A*-deficient tumors, EZH2 loss may paradoxically accelerate tumor progression. Therefore, clinical development of EZH2 inhibitors in PDAC will require biomarker-guided patient stratification integrating mutational, transcriptomic, and epigenomic features to identify PRC2-dependent subsets.

Although EZH2 inhibitors such as tazemetostat have received FDA approval for EZH2 gain-of-function mutant lymphomas and SMARCB1-deficient sarcomas—where tumors exhibit oncogene addiction to aberrant PRC2 activity—their efficacy in pancreatic ductal adenocarcinoma (PDAC) remains limited and not clearly established in clinical settings. Several mechanistic factors contribute to this discrepancy. First, PDAC tumors typically show EZH2 overexpression without recurrent gain-of-function mutations, resulting in heterogeneous PRC2 dependency rather than the strict oncogenic reliance seen in approved indications; consequently, EZH2 inhibition often fails to induce robust apoptosis or growth arrest [43,77]. Second, context-dependent PRC2 function is evident in KDM6A (UTX)-deficient PDAC subsets (common in squamous/basal-like phenotypes), where loss of the H3K27me3 eraser paradoxically enhances chromatin reprogramming and super-enhancer activation; EZH2 inhibition in this background can trigger compensatory derepression of oncogenic pathways or enhancer remodeling, limiting therapeutic benefit [78]. Third, redundancy within Polycomb complexes allows EZH1 to compensate for EZH2 catalytic inhibition, sustaining H3K27me3 deposition and repressive programs in many PDAC models [43,79]. Fourth, desmoplastic barriers in PDAC severely restrict intratumoral drug penetration, preventing sustained target engagement despite systemic dosing [77]. Finally, the absence of validated predictive biomarkers—such as specific EZH2/PRC2-dependent gene signatures, H3K27me3 landscapes, or subtype-specific dependencies—precludes enrichment of responsive patients in trials [26,80].

These limitations highlight that EZH2 targeting in PDAC requires strategies beyond monotherapy. Future directions include dual EZH1/EZH2 inhibition (valemetostat) to overcome redundancy, rational combinations with BET inhibitors (to exploit enhancer vulnerabilities in KDM6A-deficient tumors), HDAC inhibitors (to reverse compensatory acetylation), or immunotherapy (to leverage EZH2-mediated immune evasion reversal), and biomarker-guided stratification using multi-omic profiling of PRC2 dependency signatures [30,77].

#### 3.1.2. CBP/p300 Acetyltransferases

CBP and p300 are transcriptional coactivators that catalyze histone H3 lysine 27 acetylation (H3K27ac), a hallmark of active enhancers and super-enhancers [81]. In PDAC, oncogenic KRAS signaling exploits CBP/p300-dependent enhancer remodeling to sustain oncogenic transcriptional programs governing proliferation, metabolic rewiring, and lineage identity [64,82].

Genome-wide enhancer profiling has revealed extensive KRAS-driven super-enhancer formation controlling expression of oncogenes such as *MYC* and *FOSL1* [83]. Pharmacological inhibition of CBP/p300 catalytic activity using compounds such as A-485 reduces global H3K27ac levels, disrupts enhancer activity, and suppresses MYC-driven transcription in PDAC models [84,85] Combined inhibition of CBP/p300 and BET proteins produces synergistic antitumor effects by dismantling enhancer–promoter communication networks essential for PDAC cell survival [86], supporting the rationale for dual epigenetic targeting strategies.

CBP/p300 are highly homologous HATs that acetylate H3K27 and other residues to activate enhancers and drive oncogenic transcription in PDAC, including stemness genes (SOX9) and stromal collagen production. Their dual bromodomain (reader) and HAT (writer) domains make them attractive targets, with inhibitors classified by mechanism:

(i) Catalytic (HAT domain) inhibitors target the acetyltransferase active site, blocking substrate acetylation and enhancer activation. The most advanced is A-485, a potent, selective HAT inhibitor with nanomolar potency that suppresses proliferation in KRAS-driven cancer models, including preclinical PDAC lines, by reducing H3K27ac at oncogenic enhancers and downregulating stemness/EMT programs. Related compounds like CCS1477 (inobrodib) inhibit CBP/p300 HAT activity indirectly via bromodomain engagement but show broader effects. Preclinical PDAC data are limited but supportive in KRAS-mutant contexts (synergy with BETi or chemo). CCS1477 is in Phase I/II trials (NCT04068597, NCT03564132) for advanced solid tumors and hematologic malignancies, with early signals in prostate and other cancers, but no PDAC-specific cohorts have been reported yet [30,87,88].
(ii) Bromodomain inhibitors antagonize the acetyl-lysine reader module, displacing CBP/p300 from chromatin and disrupting enhancer recruitment of BRD4/MYC. Key probes include SGC-CBP30 and I-CBP112, which selectively bind CBP/p300 bromodomains over other BRDs, reducing super-enhancer activity and oncogene transcription (MYC, BCL2). In PDAC models, these show emerging anti-proliferative effects, often in combination with BETi to block compensatory pathways. Activity is documented in hematologic malignancies and solid tumors (prostate, lung), with PDAC-specific evaluation preclinical and promising in organoid/xenograft models of enhancer-addicted subtypes. No bromodomain-only CBP/p300 inhibitors are in PDAC trials; CCS1477 (bromodomain-focused) advances clinical development [88,89,90].
(iii) Dual CBP/p300 inhibitors coordinate HAT and bromodomain blockade for more complete disruption of enhancer activation. These include dual BET/CBP/p300 bromodomain compounds like NEO2734 (EP31670) and XP-524, which potently suppress KRAS-driven transcriptional programs, reduce H3K27ac, and inhibit PDAC growth in vitro/ex vivo/in vivo (patient-derived xenografts, organoids). NEO2734 shows superior efficacy over single-target inhibitors in PDAC models, overcoming BET resistance and synergizing with immune checkpoint blockade. Preclinical PDAC data are robust, with strong antitumor activity in KRAS-mutant contexts. NEO2734 has entered early clinical trials in solid tumors/prostate cancer, with PDAC relevance inferred [88,91,92].
(iv) PROTACs and degraders recruit E3 ligases (CRBN/VHL) for ubiquitin-mediated proteasomal degradation of CBP/p300, achieving durable suppression beyond catalytic inhibition. Examples include QC-182 (based on CCS1477) and others (CBPD-409, MJP6412), which potently deplete CBP/p300 in solid tumor models (HCC, prostate), downregulate oncogenic transcription, and show superior antiproliferative effects. In PDAC, degradation strategies hold promise for overcoming redundancy/resistance in enhancer-driven subtypes, though data remain preclinical (KRAS-context synergy inferred). No CBP/p300 PROTACs are in clinical trials yet, but rapid progress in related degraders suggests potential [93,94,95,96].

Overall, while HAT and bromodomain inhibitors show mechanistic promise in PDAC preclinical models (especially KRAS-driven enhancer addiction), clinical translation lags behind hematologic indications due to PDAC’s stromal barriers and heterogeneity. Dual and degrader approaches may offer greater durability/selectivity. Ongoing trials (CCS1477) and biomarker development (H3K27ac signatures) will clarify PDAC utility.

### 3.2. Epigenetic Readers

#### 3.2.1. BET Proteins Family

BET proteins, BRD2, BRD3, and BRD4, recognize acetylated lysine residues and recruit transcriptional machinery to active chromatin regions, particularly super-enhancers [97]. In PDAC, BRD4 functions as a transcriptional integrator linking KRAS signaling to enhancer activation and oncogenic transcriptional output [86,98].

BET proteins drive expression of key oncogenes and survival factors, including *MYC*, *FOSL1*, and *BCL2L1* [99,100]. BET inhibitors such as JQ1, OTX015, GSK1324726A, and the dual BET/CBP inhibitor NEO2734 suppress proliferation, induce apoptosis, and reduce tumor growth in PDAC cell lines and mouse models [101,102,103]. Beyond tumor-intrinsic effects, BET inhibition modulates the tumor microenvironment by attenuating fibroblast activation and enhancing immune cell infiltration, providing a rationale for combination with immune checkpoint blockade [104].

Despite promising preclinical results, early-phase clinical trials of BET inhibitors in solid tumors have revealed dose-limiting toxicities and modest single-agent efficacy. In PDAC, their therapeutic potential likely resides in rational combinations—particularly with MEK inhibitors, HDAC inhibitors, CBP/p300 inhibitors, or immunotherapies—to overcome adaptive resistance driven by enhancer reprogramming.

#### 3.2.2. PRC1 Components: CBX Proteins and BMI1

Chromobox (CBX) family proteins recognize H3K27me3 and recruit PRC1 complexes to repressed chromatin [105]. In PDAC, CBX proteins display context-dependent functions: CBX7 maintains epithelial identity and suppresses invasion, whereas CBX4 and CBX8 promote hypoxia adaptation, EMT, and metastasis [106,107,108,109,110].

Selective inhibition of CBX chromodomains using small molecules such as UNC3866 disrupts PRC1 recruitment and transcriptional repression in preclinical models [80]. Although CBX-directed therapies remain experimental, these findings highlight PRC1 readers as potential subtype-modulating targets in PDAC.

BMI1, a core PRC1 component, is enriched in stem-like PDAC cell populations and promotes tumor initiation, chemoresistance, and metastatic potential [111]. Pharmacological inhibition of BMI1 (PTC-209) reduces sphere formation, impairs tumor growth, and sensitizes PDAC cells to gemcitabine in preclinical models [111].

RING1A/B ubiquitin ligases cooperate with BMI1 to deposit H2AK119ub, sustaining repression of differentiation-associated genes and reinforcing the basal-like transcriptional state characteristic of aggressive PDAC [112]. Although PRC1-directed therapies remain largely experimental, these findings highlight subtype-modulating vulnerabilities within Polycomb networks.

#### 3.2.3. Emerging Epigenetic Readers in PDAC

Beyond well-characterized readers such as BET proteins (BRD4), CBX chromodomains (PRC1-mediated H3K27me3 recognition), and BMI1 (PRC1 component), a diverse array of additional epigenetic reader domains contributes to PDAC chromatin dynamics, enhancer remodeling, and oncogenic programs. These include PHD fingers, Tudor domains, chromodomains, WD40 repeats, and architectural non-histone readers. While direct PDAC evidence is often emerging or inferred from related cancers, their roles in gene activation/repression, DNA damage response, splicing, and PRC2 propagation highlight therapeutic opportunities.

(i) PHD finger proteins recognize methylated lysines (H3K4me3) to regulate transcription and DNA repair. ING2 (Inhibitor of Growth 2) binds H3K4me3 via its PHD domain, recruiting HDAC complexes to repress genes and promote apoptosis/DNA damage responses; its downregulation in PDAC may contribute to survival and chemoresistance. BPTF (Bromodomain PHD Finger Transcription Factor), the largest NURF subunit, reads H3K4me3 and facilitates chromatin remodeling; BPTF depletion sensitizes PDAC cells to chemotherapy by repressing ABC transporters and multidrug resistance (MDR) pathways in preclinical models. PHD readers thus support oncogenic transcription and therapy evasion, with emerging inhibitors (fragment-based ligands for BPTF PHD) showing promise [113,114].
(ii) Tudor domain proteins bind methylated arginines/lysines to modulate splicing, transcription, and stress responses. TDRD3 recognizes asymmetric dimethylarginine (H4R3me2a) via its Tudor domain, recruiting TOP3B to resolve R-loops and promote proliferation; its overexpression correlates with poor outcomes in some cancers, with inferred PDAC relevance via arginine methylation pathways (PRMT1-driven). SMN (Survival Motor Neuron) Tudor domain binds symmetric dimethylarginine on spliceosomal proteins (Sm proteins), ensuring splicing fidelity; dysregulation may contribute to RNA metabolism alterations in PDAC. Tudor readers link arginine methylation to RNA processing and genome stability, offering potential in PRMT-inhibitor combinations [115,116]
(iii) Chromodomain proteins (beyond CBX) recognize methylated lysines/arginines for remodeling. CDY1 binds H3K9me3/H3K27me3 to repress transcription; limited PDAC data suggest roles in heterochromatin maintenance. CHD family helicases (CHD1, CHD4) contain chromodomains and ATPase activity for nucleosome sliding; CHD1 loss or mutation disrupts enhancer integrity and cooperates with KRAS in PDAC subtypes, promoting dedifferentiation/EMT. CHD readers/remodelers thus influence chromatin accessibility and subtype plasticity [117,118]
(iv) WD40 repeat proteins include EED (Embryonic Ectoderm Development) in PRC2, which binds H3K27me3 via its β-propeller domain to allosterically activate EZH2 and propagate repressive marks. EED stabilizes PRC2 spreading in PDAC, sustaining silencing of tumor suppressors; targeting EED (allosteric inhibitors like MAK683) disrupts this feedback, offering synergy with EZH2i to overcome redundancy [119,120]
(v) HMGA1 (High Mobility Group AT-hook 1), a non-histone chromatin-binding protein that functions as an epigenetic modulator and reader-like factor. HMGA1 binds preferentially to AT-rich sequences in the minor groove of DNA, inducing conformational changes that alter nucleosome positioning, enhance chromatin accessibility, and facilitate recruitment of transcriptional regulators, co-activators, and epigenetic complexes [121]. In PDAC, HMGA1 is strongly overexpressed in tumor cells compared to normal pancreatic tissue, with high levels correlating with advanced tumor grade, dedifferentiation, lymph node metastasis, and poor overall survival [122]. Overexpression activates oncogenic transcriptional programs, including upregulation of genes involved in proliferation (via COX-2 axis), survival, and chemoresistance (to gemcitabine via Akt-dependent mechanisms) [123]. Critically, HMGA1 promotes key hallmarks of PDAC progression: it drives epithelial–mesenchymal transition (EMT) through repression of epithelial markers (E-cadherin) and activation of mesenchymal programs; enhances stem-like reprogramming by sustaining cancer stem cell properties; and contributes to aggressive biology in molecular subclasses, such as the HMGA1/FGF19-overexpressing subset associated with extremely poor outcomes and enhanced stroma formation [124]. Targeting HMGA1—via direct inhibitors, siRNA, or disruption of its interactions with epigenetic partners—represents an emerging strategy to attenuate PDAC aggressiveness, particularly in high-HMGA1 subsets [125]. Future studies should explore HMGA1 as a biomarker and therapeutic vulnerability in combination with epigenetic regimens.

### 3.3. Epigenetic Erasers

#### 3.3.1. Histone Deacetylases (HDACs)

HDAC1–11 removes acetyl groups from histone tails, promoting chromatin compaction and transcriptional repression [126]. HDAC1, HDAC2, HDAC3, and HDAC6 are frequently overexpressed in PDAC and correlate with poor prognosis and therapy resistance [127,128].

HDAC inhibitors—including vorinostat, panobinostat, entinostat, romidepsin, and mocetinostat—induce apoptosis, upregulate *CDKN1A*, reverse EMT phenotypes, and enhance sensitivity to gemcitabine and radiotherapy in preclinical PDAC models [128,129]. HDAC inhibition also remodels the tumor microenvironment by reducing stromal density and modulating immune signaling [130].

HDAC11 has emerged as a regulator of metabolic adaptation and immune evasion in PDAC, and its inhibition enhances antitumor immune responses in vivo [131]. Dual targeting strategies combining HDAC inhibition with PI3K, BET, or immune checkpoint blockade demonstrate strong synergy in preclinical PDAC models [132].

However, early-phase clinical trials of HDAC inhibitors in PDAC have demonstrated limited efficacy and substantial toxicity when used as monotherapy. These findings suggest that HDAC inhibition is unlikely to succeed alone but may retain value in rational combinations with chemotherapy, PI3K/AKT pathway inhibitors, radiotherapy, or immunotherapy.

#### 3.3.2. Histone Demethylases: KDM6A/B and LSD1

KDM6A (UTX) and KDM6B (JMJD3) demethylate H3K27me3, antagonizing PRC2-mediated repression [133]. Loss-of-function mutations in *KDM6A*, present in approximately 20% of PDAC cases, promote dedifferentiation and drive basal-like transcriptional programs [19,134]. Pharmacological targeting of KDM6B using GSK-J4 suppresses inflammatory transcriptional networks and sensitizes PDAC cells to chemotherapy in vivo [135].

LSD1 (KDM1A) demethylates H3K4me1/2 and represses enhancer activity. LSD1 overexpression promotes EMT, stemness, and metastasis in PDAC [136,137]. LSD1 inhibitors restore epithelial differentiation and synergize with BET or HDAC inhibition in organoid and xenograft models [138].

#### 3.3.3. Histone Deubiquitinases (BAP1, USP7)

Histone deubiquitinases such as BAP1 and USP7 regulate ubiquitin-dependent chromatin signaling. BAP1 removes H2AK119ub and functions as a tumor suppressor by maintaining chromatin accessibility and genomic integrity [139]. BAP1 loss in PDAC promotes genomic instability and metabolic reprogramming [140].

USP7 stabilizes MDM2 and suppresses p53 activity; pharmacological inhibition of USP7 (P5091) reactivates p53 signaling and induces apoptosis in PDAC models [141]. These findings identify histone deubiquitinases as emerging therapeutic targets within the epigenetic landscape of PDAC.

A summary of the principal epigenetic regulators involved in pancreatic ductal adenocarcinoma, including their molecular functions and biological roles, is provided in Table 1.

### 3.4. Evolution of Epigenetic Inhibitors

The development of epigenetic inhibitors has evolved through distinct generations, driven by the need to overcome limitations of early agents while addressing PDAC’s complex epigenetic landscape (chromatin redundancy, adaptive rewiring, desmoplastic barriers, and subtype heterogeneity) (Figure 3).

First-generation inhibitors (DNMTi: azacitidine, decitabine; HDACi: vorinostat/SAHA, romidepsin) were pioneered in the 1960s–2000s based on broad pharmacological reactivation of silenced genes via non-selective inhibition of DNA methylation or histone deacetylation. Their rationale stemmed from observations that global epigenetic reversal could restore tumor suppressor expression and induce differentiation/apoptosis in cancer cells. These agents achieved FDA approvals in hematologic malignancies (MDS/AML for DNMTi; CTCL for HDACi) but showed limited efficacy in solid tumors like PDAC due to off-target effects, dose-limiting toxicities (myelosuppression, GI/cardiac issues), poor pharmacokinetics (short half-life, i.v. administration), and inability to sustain target engagement amid compensatory pathways [80,142].

Second-generation inhibitors focused on improved selectivity, tolerability, and drug-like properties. Examples include oral DNMTi formulations (cedazuridine-decitabine to overcome cytidine deaminase inactivation), class/isoform-selective HDACi (entinostat for HDAC1/2; tucidinostat/chidamide), and EZH2-specific inhibitors (tazemetostat). The rationale was to minimize off-target toxicity while enhancing bioavailability (oral dosing) and target specificity, enabling better combination potential. These yielded tolerability gains and approvals in select indications (tazemetostat for EZH2-mutant follicular lymphoma and epithelioid sarcoma), with preclinical PDAC data showing synergy with chemo/immunotherapy. However, monotherapy efficacy in PDAC remains modest, as partial selectivity fails to fully counteract epigenetic redundancy (EZH1 compensation for EZH2 inhibition) [30,80].

Third-generation strategies represent a paradigm shift toward precision degradation and multi-target blockade to address resistance. These include PROTACs (proteolysis-targeting chimeras) that recruit E3 ligases (CRBN/VHL) for ubiquitin-mediated degradation of targets like EZH2, BRD4/BET, or HDAC isoforms; dual inhibitors (EZH1/EZH2 co-inhibitors like valemetostat; EZH2/BRD4 dual compounds); and allosteric/non-catalytic modulators that disrupt protein–protein interactions or scaffold functions rather than enzymatic activity. The rationale is catalytic degradation (sub-stoichiometric dosing, sustained effect even at low exposure) or complete blockade of compensatory mechanisms (EZH1 bypassing EZH2i; BET-independent super-enhancers). Preclinical data show superior potency/selectivity in PDAC models (HDAC-PROTACs overcome inhibitor resistance; EZH2 PROTACs degrade non-canonical complexes; dual EZH2/BRD4 inhibitors synergize in KRAS-driven tumors) with improved pharmacokinetics and reduced toxicity compared to first/second generations [96,143,144].

While selectivity (isoform-specific), safety (lower off-targets), and pharmacokinetics (oral bioavailability, longer half-life) generally improve across generations, consistent antitumor efficacy gains in PDAC have not been clinically demonstrated. Challenges include persistent chromatin redundancy/adaptive rewiring (alternative PRC2-independent repression), lack of validated predictive biomarkers (PRC2 dependency signatures), and PDAC-specific barriers (stromal exclusion, rapid clearance). Future directions emphasize covalent inhibitors for irreversible engagement, PROTAC optimization for tissue penetration, biomarker-stratified trials (multi-omics-guided patient selection), and rational combinations (epigenetic priming + immunotherapy/chemo) to exploit vulnerabilities like viral mimicry or synthetic lethality.

**Table 1:** Epigenetic key regulators in pancreatic ductal adenocarcinoma (PDAC).

Regulator	Mechanistic Role	Functional Consequence in PDAC	Therapeutic Implication
DNMT1 (writer)	Maintenance DNA methyltransferase; adds methyl groups to hemimethylated CpG sites during replication; overexpressed	Hypermethylation of tumor suppressors (CDKN2A, SPARC, RASSF1A); promotes proliferation, chemoresistance, genomic instability [145,146]	DNMT inhibitors (azacitidine, decitabine) under investigation; potential combination with chemotherapy or immunotherapy
TET1 (eraser)	Oxidizes 5mC to 5hmC for active demethylation; frequently downregulated/loss of 5hmC	Reduced demethylation; activates EMT/Wnt/β-catenin/Hedgehog; increases invasion/metastasis/chemoresistance [147]	Restoration of TET activity or epigenetic reprogramming strategies may suppress metastasis
MeCP2 (reader)	Binds methylated CpG; recruits HDACs/Sin3A repressive complexes; context-dependent dysregulation	Aberrant repression/chromatin compaction; gene silencing/altered splicing; contributes to progression [148]	Targeting MeCP2-associated chromatin complexes; indirect targeting via HDAC inhibitors
MBD1 (reader)	Binds methylated CpG; recruits repressive machinery; overexpressed	Represses KEAP1 → NRF2 activation; antioxidant survival/resistance to oxidative stress [149]	Targeting NRF2 axis; potential vulnerability to oxidative stress–inducing therapies
MBD4 (reader)	Methyl-CpG-binding endonuclease; mismatch repair at methylated sites; reduced/mutated	Defective repair of deaminated 5mC; genomic instability/mutation burden increase [150]	Potential biomarker for DNA damage response–targeted therapies
SUV39H2 (KMT1B)	Overexpression	H3K9 trimethylation; repression of tumor suppressor genes; enhanced homologous recombination [151]	Targetable via histone methyltransferase inhibitors (emerging class)
PRMT1 (writer)	Arginine methyltransferases; methylate H3R2/H4R3 or non-histone; variable/prognostic	Regulate p14ARF/transcription/RNA processing; promote survival/proliferation; emerging in PDAC [152]	PRMT inhibitors (in development); context-dependent targeting strategies
LSD2 (KDM1B)	Upregulated	Demethylation of H3K4; promotion of proliferation and survival [153]	LSD inhibitors (e.g., tranylcypromine derivatives) as potential therapeutic approach
JMJD1A (KDM3A)	Overexpression	Activation of cell cycle genes; enhanced tumor growth [154]	Targeting Jumonji-domain demethylases (emerging small-molecule inhibitors)
UX (KDM6A) (eraser)	Demethylates repressive H3K27me3; frequently mutated/lost in PDAC	Loss impairs enhancer activation; cooperates with KRAS for squamous differentiation/progression [134]	Sensitivity to BET inhibitors; synthetic lethal strategies under exploration
HAT1 (KAT1)	Overexpression	Increased histone acetylation; upregulation of PD-L1; immune evasion [155]	Combination with immune checkpoint blockade
HDAC1/2 (eraser)	Remove acetyl groups from histones; deacetylate promoters; overexpressed	Repress TSGs (p27, p53, E-cadherin); chromatin condensation; EMT/invasion/metastasis; immune evasion [156]	HDAC inhibitors (vorinostat, panobinostat) in combination regimens
BRD4 (reader)	Bromodomain readers; bind acetylated histones; drive super-enhancers; overexpressed	Activate oncogenes (c-MYC, BCL2); PD-L1 upregulation; survival/immune evasion; stromal support [157]	BET inhibitors (JQ1, OTX015); dual BET-CBP; combinations with HDAC/EZH2i; reduce fibrosis/immune suppression
CBP/p300 (writer/reader)	HATs; acetylate H3K27/H3K18; activate enhancers; dysregulated/overexpressed in contexts	Activates stemness genes (SOX9); stromal collagen; repair genes; contributes to desmoplasia/TME remodeling [158]	Catalytic inhibitors (A-485, CCS1477); bromodomain inhibitors (SGC-CBP30); PROTACs; dual HAT/BD blockade; emerging in KRAS-driven PDAC
SIRT1 (SIR2L1)	Overexpression	Maintenance of cancer stem cell phenotype; metabolic adaptation; tumor progression [159]	Sirtuin inhibitors; targeting CSC populations
SIRT4 (SIR2L4)	Downregulated via UHRF1-mediated repression	Increased glycolysis and proliferation [160]	Targeting UHRF1–SIRT4 axis; metabolic vulnerabilities
SIRT5 (SIR2L5)	Frequently downregulated	Metabolic rewiring; promotion of KRAS-driven tumorigenesis [161]	Targeting metabolic dependencies; synthetic lethal metabolic strategies

## 4. Non-Coding RNAs and Epigenetic Crosstalk

Non-coding RNAs (ncRNAs) represent a critical regulatory layer within the epigenetic landscape of PDAC. Acting both upstream and downstream of chromatin-modifying enzymes, ncRNAs coordinate transcriptional programs by interacting with DNMTs, histone modifiers, and chromatin remodeling complexes [162]. Through these interactions, ncRNAs contribute to tumor initiation, lineage plasticity, metastatic progression, and therapeutic resistance.

### 4.1. MicroRNAs

MicroRNAs (miRNAs) regulate gene expression post-transcriptionally by targeting mRNA transcripts for degradation or translational repression. Importantly, many miRNAs directly target epigenetic regulators, thereby indirectly shaping chromatin accessibility and transcriptional states. In PDAC, the tumor-suppressive miR-34a is frequently silenced through promoter hypermethylation [163]. Restoration of miR-34a represses NOTCH1 and BCL2 signaling pathways, leading to reduced proliferation, increased apoptosis, and impaired tumor growth in preclinical models [164,165,166]. This exemplifies a bidirectional feedback loop in which epigenetic silencing of a miRNA reinforces oncogenic transcriptional networks.

Conversely, oncogenic miRNAs such as miR-21 are consistently upregulated in PDAC and are associated with poor prognosis, chemoresistance, and enhanced desmoplasia [167,168]. Mechanistically, miR-21 promotes TGF-β signaling, fibroblast activation, and immune modulation within the tumor microenvironment [169]. By influencing both stromal and tumor compartments, miR-21 contributes to epigenetic reprogramming that stabilizes aggressive transcriptional phenotypes. Collectively, miRNAs serve as modulators of epigenetic enzyme expression, forming regulatory circuits that reinforce oncogenic signaling pathways in PDAC.

### 4.2. Long Non-Coding RNAs

Long non-coding RNAs (lncRNAs) often exert more direct chromatin-level effects by functioning as molecular scaffolds that recruit histone-modifying complexes to specific genomic loci. HOTAIR, one of the most extensively studied oncogenic lncRNAs, interacts with the PRC2, facilitating H3K27 trimethylation and transcriptional silencing of tumor suppressor genes [87,170]. In PDAC, elevated Hox Transcript Antisense RNA (HOTAIR) expression correlates with enhanced invasion, metastasis, and poor clinical outcome [171], highlighting its role in maintaining repressive chromatin states associated with tumor progression.

Similarly, MALAT1 regulates chromatin accessibility and alternative splicing programs linked to EMT, metastatic dissemination, and aggressive tumor behavior in PDAC [172,173]. Through interactions with chromatin-associated proteins and transcription factors, Metastasis-Associated Lung Adenocarcinoma Transcript 1 (MALAT1) contributes to transcriptional plasticity and adaptation to microenvironmental stress.

Another lncRNA, HOXA Transcript at the Distal Tip (HOTTIP), transcribed from the HOXA locus, facilitates chromatin looping that recruits WDR5–MLL complexes to target promoters, resulting in increased H3K4 trimethylation and activation of HOXA gene expression [174]. In PDAC cells, HOTTIP overexpression enhances proliferation, invasion, and stem-like features, underscoring its function as an epigenetic driver of tumor aggressiveness [175,176]. Together, lncRNAs integrate chromatin remodeling with spatial genome organization, reinforcing oncogenic transcriptional programs in PDAC.

### 4.3. Circular RNAs

Circular RNAs (circRNAs) add another dimension to epigenetic regulation. Many circRNAs function as microRNA sponges, sequestering tumor-suppressive miRNAs and thereby indirectly increasing expression of oncogenic targets. For example, circFOXK2 promotes PDAC progression by binding tumor-suppressive miRNAs and sustaining oncogenic transcriptional programs [177]. Beyond miRNA sequestration, emerging evidence suggests that certain circRNAs interact with chromatin-associated proteins, influencing histone modification patterns and transcriptional outputs [178].

Although the mechanistic understanding of circRNA–chromatin interactions in PDAC remains incomplete, accumulating data indicate that circRNAs contribute to epigenetic plasticity and may participate in adaptive responses to therapy.

Collectively, ncRNAs function as key mediators of epigenetic crosstalk in PDAC. By regulating and being regulated by DNA methylation and histone modification pathways, ncRNAs establish multilayered feedback circuits that stabilize oncogenic transcriptional states.

This interconnected network has important therapeutic implications. Targeting ncRNA–epigenetic enzyme interactions, through antisense oligonucleotides, miRNA mimics, or lncRNA-directed inhibitors, may disrupt transcriptional plasticity, sensitize tumors to chemotherapy or immunotherapy, and reduce metastatic potential. However, challenges, including delivery efficiency, tissue specificity, and off-target effects, must be addressed before ncRNA-based therapies can be broadly implemented in PDAC.

Overall, ncRNAs represent both biomarkers and functional regulators of the epigenetic architecture in PDAC, reinforcing the concept that effective therapeutic strategies will likely require coordinated targeting of multiple epigenetic layers.

### 4.4. ncRNAs as Therapeutic Targets in PDAC

ncRNAs, including miRNAs and lncRNAs, represent dual opportunities in PDAC therapy: as actionable targets to inhibit oncogenic drivers and as tools/vectors for delivery of therapeutic payloads. Progress spans preclinical models to early clinical trials, though challenges persist in delivery, specificity, and translation.

(i) Therapeutic targeting of oncogenic ncRNAs using antisense oligonucleotides (ASOs)—Locked nucleic acid (LNA)-modified GapmeRs or ASOs degrade specific lncRNAs via RNase H recruitment. GapmeRs targeting oncogenic HOTAIR or MALAT1 reduce PDAC invasion, migration, and chemoresistance in preclinical models (xenografts, organoids) by disrupting epigenetic silencing of tumor suppressors or EMT programs. These approaches reverse lncRNA-mediated TME remodeling and enhance gemcitabine sensitivity, highlighting their potential in overcoming resistance [179,180].
(ii) miRNA replacement therapy and anti-miR approaches—Tumor-suppressive miRNAs are replaced via synthetic mimics, while oncomiRs are inhibited. The miR-34a mimic MRX34 (liposomal formulation) entered Phase I trials for advanced solid tumors (including limited PDAC cases), showing manageable toxicity and some activity via p53 pathway restoration, though development halted due to immune-related adverse events. Preclinical miR-34a mimics inhibit PDAC stemness and metastasis. Conversely, anti-miR-21 oligonucleotides suppress CAF activation, stromal remodeling, and gemcitabine resistance in PDAC models (xenografts, organoids) by derepressing targets like PDCD4/PTEN, reducing fibrosis and enhancing drug penetration [179,181,182].
(iii) CRISPR-based ncRNA targeting—RNA-targeting CRISPR systems (e.g., Cas13/CasRx) enable precise degradation of oncogenic lncRNAs without DNA editing. Cas13 screens have identified essential lncRNAs in PDAC cells, and targeted Cas13a/CasRx delivery depletes KRAS transcripts or oncogenic lncRNAs in preclinical models, modulating growth/metastasis. These approaches overcome limitations of DNA-targeting CRISPR in non-coding regions and hold promise for transient, reversible modulation [183].
(iv) ncRNA-based delivery platforms—Exosomes and nanoparticles overcome PDAC’s stromal/hypovascular barriers for targeted ncRNA delivery. MSC-derived exosomes load and deliver chemotherapeutics (paclitaxel) or ncRNAs (anti-miRs, siRNAs) to tumor sites, enhancing efficacy and reducing toxicity in PDAC models. Nanoparticle-formulated mimics/inhibitors (lipid nanoparticles) improve stability and tumor accumulation. Exosome-mediated ncRNA transfer modulates TME (CAF reprogramming, immune sensitization), with preclinical data showing reduced metastasis and improved survival [184].
(v) Clinical status, key challenges, and ongoing trials—No ncRNA-targeted therapy is approved for PDAC, but early-phase trials in gastrointestinal/solid tumors explore miRNA mimics (MRX34 legacy), ASOs, and nanoparticle delivery. Challenges include poor delivery efficiency across desmoplastic stroma, off-target effects/immunogenicity (cytokine storms with liposomal mimics), tissue specificity, and rapid clearance. Future strategies emphasize engineered exosomes/nanoparticles for homing, combination with immunotherapy/chemo, and biomarker-guided selection (high-oncomiR tumors).

## 5. Epigenetic Reprogramming of the Tumor Microenvironment

Pancreatic ductal adenocarcinoma (PDAC) is distinguished by a dense desmoplastic and profoundly immunosuppressive tumor microenvironment (TME), which constitutes a major barrier to effective therapy. Beyond tumor-intrinsic alterations, epigenetic mechanisms critically regulate stromal and immune compartments, shaping tumor–stroma interactions, metabolic adaptation, and immune escape. Increasing evidence indicates that epigenetic reprogramming is not merely a consequence of PDAC progression but an active driver of microenvironmental remodeling and therapeutic resistance.

### 5.1. Cancer-Associated Fibroblasts and Stromal Remodeling

Cancer-associated fibroblasts (CAFs) represent the dominant stromal population in PDAC and are central mediators of extracellular matrix (ECM) deposition, tissue stiffness, and immune exclusion. CAF activation is stabilized by epigenetic alterations that lock cells into pro-tumorigenic transcriptional programs.

Elevated HDAC activity in CAFs promotes chromatin compaction and sustains expression of genes involved in ECM remodeling, collagen deposition, and secretion of immunosuppressive cytokines. Pharmacological HDAC inhibition has been shown in PDAC models to partially revert CAFs toward a more quiescent phenotype, reduce fibrosis, improve vascular perfusion, and enhance intratumoral drug penetration [35,97]. These findings suggest that HDAC-targeted strategies may indirectly sensitize tumors to chemotherapy by modifying stromal architecture.

BET proteins further regulate inflammatory and fibrotic transcriptional networks in both tumor cells and stromal fibroblasts. BET inhibition suppresses CAF-derived cytokines such as IL-6 and CXCL12, disrupts paracrine tumor–stroma signaling, and attenuates ECM organization. In preclinical PDAC models, BET inhibitors reduce stromal desmoplasia and increase infiltration of cytotoxic CD8^+^ T cells, highlighting their capacity to reshape both structural and immune components of the TME [185,186]. Together, these observations position chromatin regulators as key determinants of stromal plasticity in PDAC.

### 5.2. Epigenetic Control of Tumor Immunogenicity

Epigenetic dysregulation within tumor cells directly contributes to immune evasion. DNA methylation–mediated silencing of antigen-processing and presentation genes, including components of the MHC class I machinery, reduces tumor immunogenicity and limits recognition by cytotoxic T cells. In parallel, hypermethylation of interferon-stimulated genes dampens type I and II interferon signaling, weakening both innate and adaptive immune responses [187,188].

DNA methyltransferase inhibitors (DNMTi) can partially reverse these effects. By inducing demethylation of silenced immune genes and reactivating endogenous retroelements, DNMTi trigger a “viral mimicry” response characterized by double-stranded RNA sensing and interferon activation. This process enhances antigen presentation and increases susceptibility to immune-mediated killing. Such immune-priming effects provide a mechanistic rationale for combining DNMTi with immune checkpoint blockade in PDAC.

Epigenetic programs also shape the differentiation and function of immune cells within the PDAC microenvironment. Tumor-associated macrophages (TAMs) frequently adopt an immunosuppressive, M2-like phenotype that supports tumor growth and fibrosis. HDAC and BET inhibition have been shown to modulate macrophage polarization, promoting a shift toward more pro-inflammatory phenotypes and enhancing anti-tumor activity [189].

Similarly, epigenetic therapies can influence regulatory T-cell (Treg) stability and effector T-cell persistence. Preclinical studies demonstrate that chromatin-modifying agents may reduce Treg-mediated suppression while augmenting cytotoxic T-cell responses, thereby partially overcoming the immune-excluded phenotype characteristic of PDAC.

These findings underscore the bidirectional nature of epigenetic regulation in the TME: tumor cells shape immune behavior through epigenetic silencing, while immune and stromal cells undergo parallel chromatin reprogramming that sustains immunosuppression.

### 5.3. Epigenetic Strategies to Overcome Immune Evasion

PDAC is notoriously resistant to immune checkpoint inhibitor (ICI) monotherapy due to its “cold” TME: low T-cell infiltration, dense desmoplasia, immunosuppressive CAFs, myeloid-derived suppressor cells (MDSCs), regulatory T cells (Tregs), and downregulated antigen presentation. Epigenetic modulators offer a powerful priming strategy by reprogramming tumor cells and the TME to restore immunogenicity.

(i) DNMTi-mediated viral mimicry—Low-dose DNMTi (azacitidine, decitabine) induces global demethylation, reactivating endogenous retroelements (EREs) and transposable elements. This generates double-stranded RNA (dsRNA) species sensed by cytosolic innate immune pathways (MDA5/RIG-I), triggering type I interferon (IFN) responses, upregulation of MHC class I machinery, and expression of cancer-testis antigens. In PDAC models, DNMTi sensitize tumors to anti-PD-1/PD-L1 by converting “cold” to “hot” states, increasing CD8^+^ T-cell infiltration and reducing immune exclusion [30,43,190].
(ii) HDACi-mediated immune sensitization —HDACi (vorinostat, entinostat) upregulate MHC class II, co-stimulatory molecules (CD80/CD86), and immunogenic cell death markers (calreticulin, HMGB1), enhancing antigen presentation and T-cell priming. HDACi also reprogram TAMs toward pro-inflammatory M1 phenotypes, reduce Treg accumulation, and disrupt CAF-mediated immunosuppression. In PDAC, combining HDACi with MEK inhibitors stabilizes GATA6-dependent MHC-I expression, preserving epithelial phenotypes, boosting CD8^+^ infiltration, and improving survival in preclinical models [191].
(iii) BET inhibition in the TME—BETi (JQ1, ZEN003694) suppresses PD-L1 expression on tumor cells and CAFs, reduces secretion of immunosuppressive cytokines (IL-6, IL-10), and decreases CAF activation/fibrosis. This promotes CD8^+^ T-cell infiltration and disrupts the immunosuppressive niche. Preclinical PDAC data show BETi synergizes with ICIs by remodeling enhancers and reversing exhaustion [76].
(iv) Epigenetic priming strategies—Low-dose combinations (DNMTi + HDACi) or sequential regimens prime PDAC for ICIs. Preclinical orthotopic models demonstrate synergistic immune activation, prolonged survival, and TME remodeling (e.g., reduced MDSCs/Tregs, increased IFN signatures). Ongoing/early trials evaluate azacitidine + pembrolizumab (NCT03264404, Phase II in advanced PDAC) and similar combinations, with signals of safety and modest activity in chemo-refractory cases.
(v) lncRNA-mediated immune regulation—Oncogenic lncRNAs like HOTAIR and MALAT1 suppress IFN signaling, promote CAF activation, and drive immune exclusion via epigenetic silencing of chemokines/antigens. Targeting these (e.g., ASOs/GapmeRs) derepresses immune pathways and augments ICI responsiveness in preclinical PDAC models [180].
(vi) Key challenges—Patient selection (e.g., subtype-specific dependencies), optimal sequencing (priming before ICI), dosing to avoid toxicity/resistance, and biomarkers (e.g., IFN signatures, MHC-I expression, ERE reactivation) remain hurdles. Heterogeneity and stromal barriers limit responses.

## 6. Limitations and Challenges of Epigenetic Inhibitor Therapies in PDAC

Despite promising preclinical data and mechanistic rationale, epigenetic inhibitors—such as DNMTi (azacitidine, decitabine), HDACi (vorinostat, panobinostat), BET (JQ1, OTX015), and EZH2i (tazemetostat)—have shown limited clinical efficacy in PDAC. Monotherapy trials have largely failed to demonstrate meaningful responses, and even combinations have yielded modest benefits, highlighting multiple barriers to translation [31,53,100]. These challenges include primary and acquired resistance, dose-limiting toxicities, pharmacokinetic hurdles unique to PDAC’s biology, and the absence of robust predictive biomarkers.

### 6.1. Resistance Mechanisms

Resistance to epigenetic therapies in PDAC arises from both intrinsic tumor heterogeneity and adaptive responses. Primary resistance stems from redundant epigenetic pathways and context-dependent dependency: for instance, EZH2 inhibition often fails due to compensatory EZH1 activity, PRC2-independent maintenance of repressive marks, or paradoxical activation of super-enhancers in KDM6A-deficient subsets [31,100,146]. HDACi and DNMTi face similar issues, with compensatory upregulation of alternative writers (DNMT3A/B) or readers (BET proteins) bypassing target inhibition [31,129].

Acquired resistance frequently involves epigenetic rebound after treatment cessation, where transient derepression is reversed by feedback loops restoring repressive chromatin states [31,121]. Adaptive rewiring includes activation of oncogenic signaling (e.g., KRAS-driven pathways) or inflammatory circuits that sustain survival programs [100]. In preclinical models, BETi resistance emerges via enhancer remodeling or MYC-independent pathways, while DNMTi resistance is linked to cytidine deaminase (CDA)-mediated inactivation or reactivation of silenced repair genes [120,129]. Combination strategies (e.g., dual EZH1/2 inhibition, BETi + HDACi) and PROTACs/degraders aim to circumvent redundancy by achieving more complete target suppression [82,100].

### 6.2. Toxicity Profiles

First-generation epigenetic inhibitors exhibit significant off-target and on-target toxicities that limit dosing and combinatorial potential. DNMTi commonly cause hematological toxicities (myelosuppression, neutropenia, thrombocytopenia) and gastrointestinal effects (nausea, vomiting, diarrhea), restricting prolonged use in PDAC patients who often have poor performance status [31,129]. HDACi are associated with cardiac complications (QT prolongation, arrhythmias), fatigue, and gastrointestinal intolerance, which have hampered combinations with chemotherapy or radiotherapy [19,31].

BETi and EZH2i show better tolerability in some contexts but still induce fatigue, thrombocytopenia, and gastrointestinal issues; EZH2i additionally risks on-target effects in normal tissues reliant on PRC2 [63,100]. Dose-limiting toxicities often prevent achievement of sustained pharmacodynamic target engagement required for meaningful epigenome reprogramming [31,121]. Emerging second- and third-generation agents (class-selective HDACi like entinostat, oral DNMTi like cedazuridine-decitabine, or selective EZH2 degraders) offer improved profiles, while nanoparticle/prodrug formulations reduce systemic exposure and enhance tumor delivery [55,100].

### 6.3. Pharmacokinetic and Delivery Barriers Specific to PDAC

PDAC’s dense desmoplastic stroma, hypovascularity, and high interstitial pressure severely impede drug penetration, resulting in suboptimal intratumoral concentrations of epigenetic inhibitors [31,100]. This is compounded by high expression of CDA in PDAC stroma and tumor cells, which rapidly inactivates cytidine-based DNMTi (decitabine) via conversion to uridine [120]. Systemic pharmacokinetics are further challenged by the rapid clearance and poor bioavailability of many first-generation agents [31].

These barriers explain why promising preclinical activity often fails to translate clinically, as seen in trials of oral azacitidine (CC-486) that showed no prolongation of relapse-free survival in adjuvant PDAC [53]. Strategies to overcome include stroma-modulating agents (PEGPH20, focal adhesion kinase inhibitors), nanoparticle formulations for enhanced tumor accumulation, and localized delivery approaches [55,100]. Oral formulations with CDA inhibitors (cedazuridine) improve DNMTi bioavailability and are under investigation in solid tumors [129].

### 6.4. Challenges in Identifying Predictive Biomarkers for Patient Stratification

The lack of validated predictive biomarkers remains a major hurdle, as epigenetic dependencies vary widely across PDAC subtypes (classical vs. basal-like) and molecular contexts [31,100]. No single marker reliably predicts response to DNMTi, HDACi, BETi, or EZH2i in PDAC; for example, EZH2 overexpression correlates with poor prognosis but not consistently with EZH2i sensitivity due to compensatory mechanisms [100,146]. Heterogeneity in PRC2 dependency, enhancer landscapes, and methylation patterns further complicates stratification [121].

Emerging approaches include multi-omic profiling (H3K27me3 signatures for EZH2i, super-enhancer maps for BETi) and pharmacodynamic readouts (global 5hmC restoration for DNMTi) [31,100]. Liquid biopsy-based detection of epigenetic marks (e.g., cfDNA methylation) holds promise for non-invasive monitoring and selection [124]. Biomarker-guided trials and adaptive designs are essential to enrich responders and accelerate progress [53,100].

### 6.5. Emerging Strategies to Overcome Limitations

To address these challenges, future efforts focus on rational combinations (e.g., epigenetic priming with immunotherapy or chemotherapy to exploit viral mimicry and re-sensitization), next-generation selective agents (PROTACs, dual inhibitors), improved delivery (nanoparticles, stroma-targeting), and precision approaches (biomarker-driven patient selection via multi-omics) [31,100,129]. Integrating epigenetic therapies into multimodal regimens, guided by real-time pharmacodynamic monitoring, offers the best path toward clinical impact in this recalcitrant disease.

## 7. Future Directions and Clinical Perspective

Epigenetic therapy in PDAC should no longer be viewed as a broad chromatin-modifying strategy, but rather as a precision intervention targeting subtype-specific chromatin vulnerabilities. Recent advances in experimental modeling, multi-omic profiling, and computational biology are transforming the development of epigenetic therapies in PDAC. As the field shifts from descriptive epigenomics to functional targeting, precision-guided strategies are becoming essential for clinical translation.

Patient-derived organoids (PDOs) recapitulate the genetic, epigenetic, and transcriptional heterogeneity of primary PDAC tumors and have emerged as robust platforms for testing epigenetic inhibitors and rational drug combinations [192,193]. PDOs preserve subtype-specific chromatin states and transcriptional dependencies, enabling functional validation of context-dependent vulnerabilities. Complementary in vivo systems, including patient-derived xenografts (PDXs) and chorioallantoic membrane (CAM) assays, allow rapid assessment of drug efficacy, stromal remodeling, and intratumoral penetration within physiologically relevant microenvironments [194,195]. These platforms are particularly important in PDAC, where dense desmoplasia significantly limits drug delivery.

Integration of these models with RNA sequencing (RNA-seq), chromatin immunoprecipitation sequencing (ChIP-seq), and assay for transposase-accessible chromatin sequencing (ATAC-seq) enables high-resolution mapping of transcriptional rewiring, enhancer remodeling, and chromatin accessibility changes following epigenetic perturbation [64,196,197]. Given that PDAC heterogeneity is driven as much by epigenetic plasticity as by genetic diversity, such integrative approaches are critical for identifying functional dependencies.

Computational modeling is increasingly used to accelerate therapeutic discovery. Network inference, pathway analysis, and machine learning–based synergy prediction algorithms enable prioritization of epigenetic–targeted combinations before experimental validation [198,199,200]. These strategies are particularly valuable for anticipating compensatory chromatin remodeling and adaptive resistance mechanisms. Systems-level integration of methylome, histone modification profiles, and non-coding RNA signatures may help define molecularly stratified PDAC subgroups with distinct epigenetic vulnerabilities, moving the field toward biomarker-guided treatment allocation. Several classes of epigenetic inhibitors—including DNMT, HDAC, BET, and PRC2 inhibitors—have been evaluated in preclinical and early clinical studies in PDAC, as summarized in Table 2.

Despite compelling preclinical evidence, no epigenetic drug has yet received regulatory approval specifically for PDAC. Agents targeting DNMT, HDAC, BET proteins, and PRC2 have entered early-phase trials—primarily in combination regimens. Clinical translation has been limited by: (i) systemic toxicity and narrow therapeutic windows; (ii) suboptimal drug penetration within the dense stromal compartment; (iii) lack of predictive biomarkers; (iv) rapid adaptive transcriptional rewiring. Nevertheless, pharmacodynamic activity and immune-modulatory effects observed in early trials support continued development in biomarker-enriched cohorts [201,202,203,204,205].

**Table 2:** Epigenetic drugs evaluated in pancreatic ductal adenocarcinoma (PDAC).

Target	Drug Name	Preclinical Evidence	Clinical Trials	Combination Strategies
DNMT	Azacitidine	Epigenetic priming; restores tumor suppressor expression [206]	Phase I/II (combination) [207]	Gemcitabine; immune checkpoint inhibitors [208]
DNMT	Decitabine	Viral mimicry induction; chemosensitization [209,210]	Early-phase trials [211]	Platinum agents; PARP inhibitors (HRD)
HDAC	Vorinostat (SAHA)	Synergistic with gemcitabine/radiation; induces CDKN1A [212]	Early-phase solid tumor trials [213]	Chemotherapy; PI3K/AKT inhibitors [214]
HDAC	Panobinostat	Pro-apoptotic; stromal modulation [53]	Basket trials [215]	Chemotherapy; immunotherapy
HDAC	Entinostat	Reverses EMT; immune activation [216]	Early-phase GI trials	PD-1/PD-L1 blockade [217]
BET	JQ1	Suppresses MYC/KRAS-driven transcription [218]	Preclinical	MEK inhibitors; chemotherapy [76]
BET	OTX015	Antiproliferative in xenografts [219]	Early-phase trials [102,220,221,222]	Targeted agents
BET/CBP	NEO2734	Super-enhancer disruption [102,221,222]	Preclinical	HDAC inhibitors; chemotherapy
EZH2	Tazemetostat	Reactivates tumor suppressors; reduces invasion [223]	Approved in other cancers; exploratory trials [224]	Gemcitabine; BET/HDAC inhibitors
HDAC/others	Mocetinostat, Romidepsin	Induce apoptosis; chemosensitization [225]	Early-phase trials [226]	Chemotherapy; immunotherapy

Future progress in PDAC epigenetic therapy will likely depend on four central pillars:

(i) Subtype-specific targeting

Molecular stratification into classical and basal-like phenotypes should guide therapeutic selection. For example, basal-like or *KDM6A*-deficient tumors may exhibit increased sensitivity to PRC2 or LSD1 inhibition, whereas classical tumors may depend more heavily on enhancer-driven transcriptional programs.

(ii) Epigenetic priming strategies

Low-dose DNMT or HDAC inhibition can sensitize PDAC cells to chemotherapy by restoring apoptotic pathways, reversing EMT, and altering DNA damage responses. Such “priming” regimens may be particularly effective in neoadjuvant or first-line metastatic settings.

(iii) Targeted and signaling combinations

Simultaneous targeting of KRAS signaling (MEK/ERK inhibition) and downstream epigenetic effectors (BET or CBP/p300 inhibitors) may achieve more durable suppression of oncogenic transcriptional networks. Similarly, combining chromatin-modifying agents with DNA damage response inhibitors (PARP inhibitors in HRD contexts) may exploit replication stress vulnerabilities.

(iv) Integration with immunotherapy

DNMT and HDAC inhibitors can enhance antigen presentation, induce interferon signaling, and remodel stromal and myeloid compartments. Combining epigenetic agents with immune checkpoint blockade represents a compelling strategy to convert immunologically “cold” PDAC tumors into more inflamed phenotypes. However, improved biomarker-driven patient selection and optimized sequencing will be essential.

Epigenetic therapies are uniquely positioned to address multiple barriers simultaneously by restoring differentiation states, increasing immunogenicity, and modulating stromal and immune compartments. However, redundancy within chromatin-regulatory networks suggests that systematic targeting of multiple epigenetic nodes, rather than single-agent approaches, will be required to achieve durable responses.

## 8. Conclusions

Epigenetic dysregulation represents a fundamental organizing principle of pancreatic ductal adenocarcinoma (PDAC) biology. Aberrant activity of epigenetic writers, readers, and erasers, including DNA methyltransferases, Polycomb complexes, histone acetyltransferases and deacetylases, BET proteins, demethylases, and deubiquitinases, establishes a highly plastic chromatin landscape that governs transcriptional subtype identity, epithelial–mesenchymal transition, stemness, immune evasion, and therapeutic resistance. Through coordinated alterations in DNA methylation, histone modifications, and non-coding RNA networks, PDAC cells acquire the capacity to dynamically adapt to metabolic, microenvironmental, and treatment-induced stress.

Importantly, this epigenetic plasticity, while contributing to the aggressiveness and refractoriness of PDAC, also creates therapeutic vulnerabilities. Preclinical studies consistently demonstrate that targeting chromatin regulators can re-sensitize tumors to chemotherapy and radiotherapy, collapse oncogenic enhancer programs, restore differentiation states, and remodel the tumor microenvironment to enhance anti-tumor immunity. These findings establish epigenetic regulators not merely as biomarkers of disease progression, but as functional drivers and actionable nodes within PDAC signaling networks.

Nevertheless, the clinical impact of first-generation epigenetic agents has been modest. Key barriers include extreme desmoplasia limiting drug delivery, profound intratumoral heterogeneity, rapid adaptive transcriptional rewiring, and the historical use of epigenetic drugs as late-line monotherapies in unselected patient populations. These challenges underscore that epigenetic therapy in PDAC cannot rely on empirical application but instead requires precision-guided strategies.

Future progress will depend on three central pillars: (i) Improved pharmacology and delivery—Development of next-generation, more selective inhibitors and stromal-penetrating formulations capable of achieving sustained intratumoral target engagement. (ii) Biomarker-guided patient stratification—Integration of genomic, epigenomic, transcriptomic, and spatial profiling to identify tumors truly dependent on specific chromatin regulators. (iii) Rational combination strategies—Embedding epigenetic modulators within multidimensional treatment regimens, including chemotherapy, KRAS-pathway inhibition, DNA damage response targeting, and immune checkpoint blockade.

## Data Availability

No new data were created or analyzed in this study.

## Abbreviations

The following abbreviations are used in this manuscript:

ADAMTS1	A disintegrin and metalloproteinase with thrombospondin motifs 1
ADP	Adenosine diphosphate
ARID1A	AT-rich interaction domain 1A
ASCO	American society of clinical oncology
ATAC	Assay for transposase-accessible chromatin
ATP	Adenosine triphosphate
BAP1	BRCA1-associated protein 1
BCL2L1	BCL2-like 1BAP1—BRCA1 associated protein 1
BCL2	B-Cell lymphoma 2
BCL2L1	BCL2-like 1
BET	Bromodomain and extra-terminal
BMI1	B lymphoma Mo-MLV insertion region 1 homolog
BNC1	Basonuclin 1
BRCA1/2	Breast cancer gene 1/2
BRD2/3/4	Bromodomain containing protein 2/3/4
CAF	Cancer-associated fibroblast
CAM	Chorioallantoic membrane
CBP	CREB-binding protein
CBX	Chromobox protein
CDH1	Cadherin 1
CDKN1A	Cyclin-dependent kinase inhibitor 1A
CDKN2A	Cyclin-dependent kinase inhibitor 2A
CREBBP	CREB binding protein
cfDNA	Cell-free DNA
CRISPR	Clustered regularly interspaced short palindromic repeats
CXCL12	C-X-C motif chemokine ligand 12
DMR	Differentially methylated region
DNA	Deoxyribonucleic acid
DNMT	DNA methyltransferase
DUB	Deubiquitinase
ECM	Extracellular matrix
EMT	Epithelial–mesenchymal transition
EP300	E1A-binding protein p300
EZH2	Enhancer of zeste homolog 2
FOLFIRINOX	5-fluorouracil, leucovorin, irinotecan, oxaliplatin
GATA6	GATA-binding protein 6
H3K27ac	Histone H3 lysine 27 acetylation
H3K27me3	Histone H3 lysine 27 trimethylation
H3K4me3	Histone H3 lysine 4 trimethylation
H3K36me2/3	Histone H3 lysine 36 di-/trimethylation
HAT	Histone acetyltransferase
HDAC	Histone deacetylase
HMT	Histone methyltransferase
HR	Homologous recombination (repair)
JMJD3	Jumonji domain-containing protein 3
KDM	Lysine demethylase
KDM1A (LSD1)	Lysine-specific demethylase 1
KDM6A/B	Lysine demethylase 6A/6B
KRAS	Kirsten rat sarcoma viral oncogene homolog
lncRNA	Long non-coding RNA
LSD1	Lysine-specific demethylase 1
miRNA	MicroRNA
ncRNA	Non-coding RNA
PARP	Poly(ADP-ribose) polymerase
PD-1	Programmed cell death protein 1
PD-L1	Programmed death-ligand 1
PDAC	Pancreatic ductal adenocarcinoma
PRC1/PRC2	Polycomb repressive complex 1/2
PTM	Post-translational modification
TME	Tumor microenvironment
TP53	Tumor protein p53
USP7	Ubiquitin-specific-processing protease 7
WGBS	Whole-genome bisulfite sequencing
