# Correction: Kubiak, K.; Inkielewicz-Stępniak, I. Epigenetic Regulators as Therapeutic Targets in Pancreatic Ductal Adenocarcinoma. *Cancers* 2026, *18*, 1001

**DOI:** 10.3390/cancers18132108

**Published:** 2026-06-29

**Authors:** Klaudia Kubiak, Iwona Inkielewicz-Stępniak

**Affiliations:** Department of Pharmaceutical Pathophysiology, Medical University of Gdansk, 80-211 Gdansk, Poland; iwona.inkielewicz-stepniak@gumed.edu.pl

Figure 1 Legend

In the original publication [1], there was a mistake in the legend for “Schematic overview of epigenetic dysregulation in PDAC”. The published figure legend inadvertently included extended explanatory text originating from the main manuscript body, rather than a concise description appropriate for a figure legend. In addition, acknowledgement of the conceptual contribution to the figure design by Maria Wiese was unintentionally omitted. The correct legend appears below. 

“The figure illustrates major epigenetic mechanisms contributing to transcriptional dysregulation and disease progression in PDAC. (**A**) DNA methylation dysregulation: In normal cells, CpG islands at promoters are unmethylated, allowing active transcription of tumor-suppressor genes. In PDAC, aberrant promoter hypermethylation silences key tumor suppressors (e.g., CDKN2A/p16, SPARC, RASSF1A), promoting proliferation and invasion. Concurrent global hypomethylation activates repetitive elements, drives genomic instability, and contributes to oncogenic transcription. These changes reinforce basal-like/squamous subtype identity and chemoresistance. (**B**) Non-coding RNA involvement: Oncogenic lncRNAs (HOTAIR recruits PRC2 for H3K27me3 deposition and gene silencing; MALAT1 modulates splicing and chromatin looping) promote EMT, metastasis, and stemness. Tumor-suppressive miRNAs (miR-34a) are frequently silenced by promoter methylation, leading to unchecked Notch/Snail signaling and progression. (**C**) Transcriptionally active (euchromatic) states: Histone acetyltransferases (HATs) (e.g., CBP/p300) add acetyl groups (H3K27ac, H4Kac) at enhancers/promoters, recognized by BET proteins to recruit transcriptional machinery. Activating methylation marks (H3K4me3, H3K36me3) are maintained by writers; lysine demethylases (KDMs) (KDM6A/UTX removes repressive H3K27me3 at enhancers; KDM1A/LSD1 demethylates H3K4me at poised states) and deubiquitinases (DUBs) (USP22 removes H2Bub1 to facilitate elongation) actively sustain open chromatin for epithelial gene programs in classical PDAC subtypes. (**D**) Transcriptionally repressed (heterochromatic) states: Polycomb repressive complexes (PRC2 via EZH2 deposits H3K27me3; PRC1 adds H2AK119ub) compact chromatin and silence differentiation genes (CDH1/E-cadherin, CDKN1A/p21), driving dedifferentiation, EMT, and basal-like phenotypes in PDAC. Histone deacetylases (HDACs) remove acetyl groups, reinforcing repression. These alterations enable rapid adaptation to therapeutic stress, subtype plasticity, immune evasion, and stromal remodeling in PDAC. Figure concept courtesy of Maria Wiese, Department of Pediatrics and Adolescent Medicine, Medical University of Göttingen, Germany. This figure was created with BioRender.com.”

The following text was mistakenly included in the original figure legend and should instead appear in the main text of the manuscript: “While epigenetic dysregulation has long been recognized as a key driver of PDAC biology beyond genetic mutations, recent reviews have advanced our understanding of specific facets. For example, Pandey et al. (2023) provided a broad overview of epigenetic machinery components in PDAC initiation and development, while Elrakaybi et al. (2022) and Sultana et al. (2025) highlighted epigenetic impacts on biology, diagnostics (methylation-based biomarkers), and emerging clinical applications [26–28]. More focused works include those by Tost et al. (2025), on leveraging epigenetic alterations for prognostic and therapeutic insights, Maietta et al. (2025), on harnessing epigenetic inhibitors to overcome PDAC treatment challenges, and Drndakova et al. (2026), on epigenetic modulation to reverse immune suppression in the tumor microenvironment [29–31]. Earlier contributions, such as methylation-centric analyses [32,33] and histone-focused or therapeutic overviews [34], laid the foundational knowledge.”

The authors would like to add the following to the Acknowledgments section.

“**Acknowledgments:** The authors gratefully acknowledge Maria Wiese (Department of Pediatrics and Adolescent Medicine, Medical University of Göttingen, Germany) for her contribution to the conceptual design of Figure 1.”

The authors state that the scientific conclusions are unaffected. This correction was approved by the Academic Editor. The original publication has also been updated.
